# Morphological and genomic comparisons of Hawaiian and Japanese Black-footed Albatrosses (*Phoebastria nigripes*) using double digest RADseq: implications for conservation

**DOI:** 10.1111/eva.12274

**Published:** 2015-06-13

**Authors:** Elisa G Dierickx, Allison J Shultz, Fumio Sato, Takashi Hiraoka, Scott V Edwards

**Affiliations:** 1 Department of Organismic and Evolutionary Biology and Museum of Comparative Zoology, Harvard UniversityCambridge, MA, USA; 2 Department of Zoology, University of CambridgeCambridge, UK; 3 Yamashina Institute for OrnithologyAbiko, Japan

**Keywords:** Black-footed Albatross, conservation genomics, double digest RADseq, gene flow, integrative taxonomy, Izu-Torishima, Midway Island, *Phoebastria nigripes*, population differentiation, subspecies

## Abstract

Evaluating the genetic and demographic independence of populations of threatened species is important for determining appropriate conservation measures, but different technologies can yield different conclusions. Despite multiple studies, the taxonomic status and extent of gene flow between the main breeding populations of Black-footed Albatross (*Phoebastria nigripes)*, a Near-Threatened philopatric seabird, are still controversial. Here, we employ double digest RADseq to quantify the extent of genomewide divergence and gene flow in this species. Our genomewide data set of 9760 loci containing 3455 single nucleotide polymorphisms yielded estimates of genetic diversity and gene flow that were generally robust across seven different filtering and sampling protocols and suggest a low level of genomic variation (θ per site = ∼0.00002–0.00028), with estimates of effective population size (*N*_e_ = ∼500–15 881) falling far below current census size. Genetic differentiation was small but detectable between Japan and Hawaii (*F*_ST_ ≈ 0.038–0.049), with no *F*_ST_ outliers. Additionally, using museum specimens, we found that effect sizes of morphological differences by sex or population rarely exceeded 4%. These patterns suggest that the Hawaiian and Japanese populations exhibit small but significant differences and should be considered separate management units, although the evolutionary and adaptive consequences of this differentiation remain to be identified.

## Introduction

Given limited resources for nature conservation, it is important to develop criteria for the prioritization of species protection efforts and a taxonomy that adequately reflects underlying genetic diversity. The concepts of evolutionary significant units (ESUs) and management units (MUs) were proposed nearly 30 years ago (Ryder 1986) as heuristics to guide such efforts. Definitions of the ESUs and MUs vary depending on the biological and legislative context, but in general, an ESU recognizes that the focal population has been isolated in the past and is therefore likely to possess distinct evolutionary potential. Additional criteria for recognition of ESUs emphasize the history of selection of genetic variation and the potential for future adaptation (Crandall et al. 2000). By contrast, recognizing MUs typically involves understanding the extent of allele frequency differences and the importance of demographic distinctness between units (Moritz 1994; Palsboll et al. 2007). Both concepts evolve as new methods for quantifying neutral and adaptive genetic diversity and distinctness in the study of natural populations.

Different types of genetic data can be used to determine and delimit ESUs and MUs, each with its own set of advantages and disadvantages. Each marker will estimate the demographic history of that marker, and, as a result, findings and implications for conservation may differ from marker to marker (Fay and Wu 1999). Different markers, whether organellar, sex-linked, or autosomal, reflect different aspects of population history (Funk and Omland 2003; Chan and Levin 2005; Toews and Brelsford 2012; Yaacov et al. 2012). Additionally, different technologies yield markers of different types, such as linked sites for mtDNA and nuclear DNA sequencing of the PCR era, dominant or co-dominant fragments in methods such as AFLPs or microsatellites, or single nucleotide polymorphisms (SNPs) in the case of next-generation sequencing methods such as RADseq (Brumfield et al. 2002; Buburuzan et al. 2007; Brito and Edwards 2009; Peterson et al. 2012). Although not without controversy (Zink and Barrowclough 2008; Edwards and Bensch 2009), the field of conservation genetics and phylogeography is in general agreement that the large numbers of loci available in the nuclear genome are superior to the single locus provided by mtDNA in delimiting species and populations for conservation and for estimating historical population parameters with greater precision with increasing numbers of loci (Felsenstein 2006; Carling and Brumfield 2007; McCormack et al. 2011; Harris et al. 2014). Additionally, recent theory (Hedrick 2005; Jakobsson et al. 2013) as well as studies in nonmodel species (Ryynanen et al. 2007; Väli et al. 2008; Coates et al. 2009) and in humans (Rosenberg et al. 2002; Li et al. 2008) reveal different patterns of resolution by different markers: for example, *F*_ST_ values between pairs of populations are often lower when based on microsatellites than when based on sequence-based markers, with the latter better reflecting population subdivision when it exists or being less constrained due to heterozygosity or statistical effects.

Conservation genetics has adopted different methods for assaying and analysing genetic variation, and has historically grappled with the different implications of each method for discovering neutral or adaptive variation, as well as optimal sampling schemes for studying endangered populations. A recent approach that takes advantage of next-generation sequencing technology is restriction site-associated DNA sequencing (RADseq; Baird et al. 2008; Etter et al. 2011; Narum et al. 2013) and its refinements (Peterson et al. 2012), which use restriction enzymes to digest throughout the genome and then sequence inwardly from the cut sites of each fragment. RADseq offers a number of tangible advantages over other genotyping techniques, making it a useful tool for delimiting ESUs and for resolving conservation questions. The large number of genotyped markers, usually in the thousands, reflects the evolutionary history of the whole genome. Furthermore, it does not require the design of species-specific primers and hence, no prior sequence knowledge about the genome of the focal species is needed. As a result, RADseq is particularly suitable for endangered and nonmodel species (Catchen et al. 2013a,b; Hohenlohe et al. 2013; Rheindt et al. 2013; Senn et al. 2013). Another advantage of RADseq is that, because of the high number of SNPs produced, population genetic parameters and structure may require a relatively small number of individuals, depending on the demographic history (Felsenstein 2006; Carling and Brumfield 2007; Green et al. 2010; Rheindt et al. 2013), making it particularly relevant for species that are rare or difficult to access. Finally, the cost of RADseq is relatively low and individuals can be multiplexed in a single sequencing lane, facilitating the sampling of multiple populations across broader geographical ranges. Taking all these factors into consideration, RADseq appears particularly well suited for population genetic studies of threatened and endangered species.

The Black-footed Albatross (*Phoebastria nigripes*; Audubon 1839) is a seabird endemic to the North Pacific, with an age of first breeding of 6–7 years and a demographic generation time of approximately 18 years (Niel and Lebreton 2005; BirdLife International 2014). After fledging and in the nonbreeding season, individuals range throughout the North Pacific Ocean, but those individuals that are resighted display strong nest fidelity and natal philopatry upon reaching reproductive maturity and in subsequent breeding seasons (Cousins and Cooper 2000). Currently, the estimated census size is 66 140 pairs, with 98% of the population breeding in the Hawaiian archipelago, 1.9% in Japan and 0.1% on offshore Mexican islands (Cousins and Cooper 2000; Flint 2007; Naughton et al. 2007; BirdLife International 2014).

In the past 200 years, the species has suffered from a number of threats: feather and egg collecting in the late 1800s and early 1900s, high-sea driftnet by-catch between 1978 and 1992, and, most recently, long line fisheries. Today, the population size of the Black-footed Albatross has stabilized thanks to a series of mitigation measures due to the species’ ‘Vulnerable’ status on the IUCN Red List of Threatened Species (Cousins and Cooper 2000; Lewison and Crowder 2003; BirdLife International 2014). As a result of these successes, in 2013, the species was downlisted from Vulnerable to Near-Threatened (IUCN 2013). However, natural phenomena predicted as consequences of climate change such as storms, rising sea levels (Storlazzi et al. 2013) and volcanic eruptions (Harrison 1990) will likely threaten the species’ breeding grounds. Despite current breeding ground protection against anthropological threats (Naughton et al. 2007), little can be performed against these natural phenomena. However, Hawaii and Japan might be affected differently by rising sea levels and increased frequency of storms. Recent climatic events have already made apparent the vulnerability of the Hawaiian islands. In 2011, a 1.5 m wave completely submerged Spit Island in Midway Atoll, killing 110 000 Black-footed and Laysan Albatross chicks and 2000 adults (Hutcherson 2011). Indeed, these islands, where 98% of the Black-footed Albatrosses breed, are only 2–5 m above sea level (Naughton et al. 2007). The Japanese and Mexican islands have higher elevations and might be less at risk of being affected by the predicted consequences of climate change. In Japan, Izu-Torishima has an elevation of 394 m, Haha-jima of 462 m and Senkaku of 383 m. Guadalupe Island off the coast of Mexico has an elevation of 1297.5 m (Google Maps 2014[Bibr b41]).

With this in mind, Black-footed Albatross conservation action plans (COSEWIC 2006, Naughton et al. 2007) recommend that efforts be made to develop the smaller albatross colonies outside of Hawaii and to encourage the natural colonization of new islands such as Wake and Guadalupe. However, these measures might need to be altered if there is underlying population structure among the breeding grounds. As a result, it is important to know whether the two largest populations located in Hawaii and Japan are genetically and morphologically distinguishable as has been previously suggested (Cousins and Cooper 2000; Walsh and Edwards 2005).

In May 2013, the Agreement on the Conservation of Albatrosses and Petrels’ (ACAP) taxonomy working group rejected a petition for these Japanese and Hawaiian populations to be considered separate subspecies due to a lack of supporting data (ACAP 2013). Morphologically, the Japanese Black-footed Albatrosses are thought to be smaller than their Hawaiian counterparts (Cousins and Cooper 2000), but this notion seems to solely rest on a personal communication that spread through the literature without strong supporting evidence. Molecular studies of genetic variation in Black-footed Albatrosses have come to varied conclusions regarding levels of genetic variation, levels of gene flow and the direction of gene flow, depending on the markers used and sampling scheme. One study involving mtDNA (Walsh and Edwards 2005) suggested significant differentiation between Japan and Hawaii, negligible gene flow and a higher genetic diversity in Hawaii. On the other hand, Eda et al. 2008 found that populations within Japan, from Izu-Torishima and the Bonin Islands, did not differ significantly in mtDNA and that the Bonin Islands had levels of genetic diversity similar to those found in Hawaii. Most recently, based on data from 10 microsatellite markers, Ando et al. (2014) suggested that birds from Hawaii and Japan are genetically differentiated and have similar levels of genetic diversity, but that there is strong asymmetrical gene flow from Japan to Hawaii. Given these diverse conclusions, additional studies with new types of data are warranted.

In the current project, we investigate the seeming contradictions between these previous studies using a comprehensive nuclear data set obtained through double digest RADseq. For the reasons discussed above, we argue that this methodology can provide more reliable population parameter estimates for conservation purposes than other markers. Our principal aim, partially motivated by the need to provide the ACAP with the additional information required to determine the phylogenetic status of the Japanese population, is to resolve whether and to what extent the Hawaiian and Japanese populations of the Black-footed Albatross represent two distinct populations. Based on previous studies and high levels of breeding philopatry (Cousins and Cooper 2000), our hypothesis is that the Hawaiian and Japanese populations of the Black-footed Albatross are clearly morphologically and genetically distinct. Genetically, we predict significant differentiation with low levels of gene flow. Morphologically, we predict the Japanese population to be significantly smaller than their Hawaiian counterparts, as has been previously implied (Cousins and Cooper 2000; Walsh and Edwards 2005). Our alternative hypothesis is that the Hawaiian and Japanese populations are panmictic or nearly so, showing high levels of gene flow between the island groups. A study by Friesen et al. (2007) found that populations sharing the nonbreeding range (as is the case for the Black-footed Albatross) are less likely to be genetically differentiated. Given the extensive foraging range of the Black-footed Albatross, during both breeding and nonbreeding periods (Cousins and Cooper 2000), it is likely that a few individuals per generation may join a colony other than their natal colony. The recent founding of colonies on Guadalupe Island and Wake Island (COSEWIC 2006) supports this idea. However, it is not clear whether these occasional events constitute enough gene flow to maintain panmixia. In contrast to the findings of Ando et al. (2014), if gene flow exists between Japan and Hawaii, we would expect it to be mainly from Hawaii towards Japan, as the Hawaiian population is much larger than the Japanese population. The Hawaiian population is statistically more likely to produce immigrants, and any alleles brought in by Japanese birds would have less impact on the Hawaiian gene pool than vice versa.

Albatrosses have been the subject of a number of population genetic studies, mostly involving mtDNA, microsatellites and AFLPs (Burg and Croxall 2001; Abbott and Double 2003a,b; Friesen et al. 2007; Milot et al. 2007, 2008). One paradigm emerging from these studies is that the high rates of natal philopatry often appear inconsistent with rates of gene flow as measured by molecular markers (Edwards et al. 2001; Friesen et al. 2007; Milot et al. 2008). We suggest that the RADseq approach is useful to investigate these questions because it reflects variation across the whole genome, arguably better than the small number of microsatellite markers often used (Zink 2010). Indeed, results based on RADseq and microsatellites have been known to differ. For example, Lozier (2014) compared the levels of genetic diversity of two species of American bumble bees, the common *Bombus impatiens* and the declining *Bombus pensylvanicus*, and his RADseq data suggest that levels of genetic diversity in these two species are in fact much more similar than previous microsatellite analyses had indicated. The current project can hence also serve as a model for how new methods can be used to complement and advance knowledge based on older methods. In addition to our genetic investigation, we took an integrative taxonomic approach by using museum specimens of known provenance to examine the claim that Japanese Black-footed Albatrosses are smaller than their Hawaiian counterparts (Cousins and Cooper 2000; Walsh and Edwards 2005). Our morphological comparison of the two breeding populations complements the genetic data obtained through RADseq and sheds light from a different perspective on the question. By combining these molecular and non-molecular methods, we were able to take an integrated approach to the taxonomy of the Black-footed Albatross.

## Materials and methods

### Field and laboratory work

**Figure 1:**
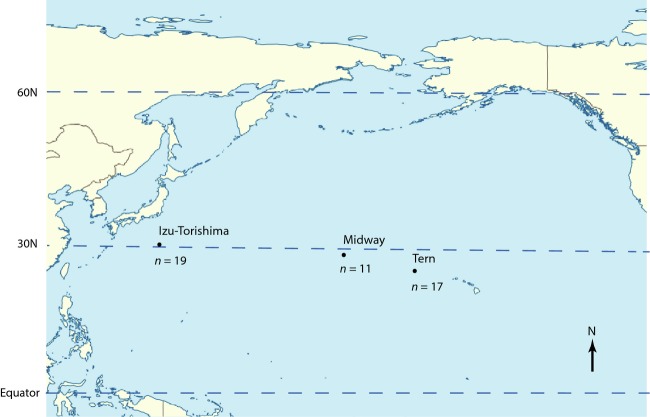
Sampling locations of the three albatross colonies. Sample sizes of sequenced individuals for each population are given below each point.

We non-destructively collected blood samples from 47 adult Black-footed Albatrosses (Table S1) in three different breeding areas, during the breeding period: 19 in 1996 from Izu-Torishima Island, sometimes also referred to as Torishima, (Japan, 30°28′N140°18′E), 11 in 1994 from Midway Island (Hawaii, 28°12′N177°21′W) and 17 in 2000 from Tern Island (Hawaii, 23°52′N166°17′W) (Fig.1). Samples were preserved in Queen’s lysis buffer (Seutin et al. 1991) or TE and stored at −80°C. Because high levels of gene flow within the Hawaiian islands have been observed in every previous study, we elected to sample only two populations within Hawaii. This high level of gene flow within the Leeward Hawaiian Islands group (Ando et al. 2014) suggests that sampling more densely may not be much more informative to detect genetic differentiation within Hawaii. Our main focus is on the presence and magnitude of differentiation, as well as levels of gene flow between Hawaii and Japan, a conclusion that is unclear based on previous studies. Although sampling additional populations would no doubt refine our understanding of genetic differentiation in this species, we suggest that it will not substantially change estimates of differentiation between Hawaii and Japan. Additionally, we believe that the ratio of the number of markers used and the number of demographic parameters to be estimated has been unacceptably low in previous studies. For example, with 10 microsatellite loci, Ando et al. (2014) purported to estimate 30 unidirectional estimates of gene flow as well as six estimates of population diversity, for a total of 36 parameters. While our study falls on the opposite end of the spectrum (3000–9000 markers for a maximum of nine parameters), we believe our sampling scheme presents a more robust, or at least an alternative, framework for estimating broad demographic parameters in this and other species.

DNA was extracted from the blood samples using the Qiagen DNeasy (Qiagen, Venlo, the Netherlands) and Omega E.Z.N.A (Omega Bio-Tek, Norcross, GA, USA). DNA extraction kits, following the manufacturers’ protocols for blood samples, except for an increased enzyme digestion time (overnight). DNA concentrations were measured with fluorometric quantification (Qubit 2.0 HS DNA assay; Invitrogen, Life Technologies, Carlsbad, CA, USA). Double digest RAD sequencing libraries for each individual sample were prepared according to the protocol of Peterson et al. (2012), using enzymes *SphI* and *EcoR1*. Each Black-footed Albatross individual was assigned a 5-base pair (bp) inline barcode, and equimolar concentrations of eight uniquely barcoded individuals were pooled and double-indexed by 16 cycles of high-fidelity PCR (KAPA Hot Start Long Range PCR kit; KAPA Biosystems, Wilmington, MA, USA) with Illumina barcodes, resulting in a unique combination of inline and Illumina barcodes for each individual. The PCR products were pooled in equimolar quantities and sequenced in one lane of a rapid run of an Illumina HiSeq 2500 sequencer at the Bauer Core facility at Harvard University, producing 100-bp paired-end reads.

### Sequence processing and alignment

We used Geneious 6.1.7 (Biomatters 2013) to sort sequence reads by barcode and trim the restriction sites; we explored different strategies for trimming the sequences and found that they made no difference for the results. The sequences were then processed with the program *process_radtags* in Stacks 1.06 (Catchen et al. 2011, 2013b). The paired-end reads were then merged into a single file per individual, before a *de novo* assembly was performed with *denovo_map.pl* in Stacks using parameters –m 3 –M 3 –n 3 –p 2. At this stage, we eliminated four individuals (two from Izu-Torishima, one from Midway, one from Tern) due to low sequence coverage. Finally, we used the *populations* module in Stacks to create data matrices (referred to as ‘data sets’, Tables S2 and S3) for subsequent analyses, with a minimum stack depth of 30. To study the effects on genetic estimates of different SNP filtering protocols, removal of low-frequency alleles and differences in sample sizes among populations, we analysed seven different data sets (numbered 1 through 7) produced from different SNP filtering protocols (Tables S2 and S3) with varying numbers of individuals and minor allele frequency (MAF) and heterozygosity requirements (Table1). Specifically, we varied the number of SNPs per locus (to avoid SNPs in potential high linkage disequilibrium), removal of SNPs with heterozygosity >0.75 and a MAF >0.05 (to avoid potential low-frequency SNP miscalls) and data set completeness.

**Table 1:** Description of data sets 1–7 and the single nucleotide polymorphism (SNP) filtering procedures used to create them

Data set number	Number of individuals per population	Total number of individuals	SNP Filtering	Data set completeness requirement for locus inclusion	Data set completeness	Total number of SNPs
1	Tern: 17 Midway: 11 Izu-Torishima: 19	43	None	At least 50% of individuals in at least 2 populations	80%	3455
2	Tern: 17 Midway: 11 Izu-Torishima: 19	43	1 SNP per locus	At least 50% of individuals in at least 2 populations	80%	2769
3	Tern: 17 Midway: 11 Izu-Torishima: 19	43	1 SNP per locus, loci required to have heterozygosity <0.75 and MAF >0.05	At least 50% of individuals in at least 2 populations	80%	2672
4	9	27	1 SNP per locus	At least 50% of individuals in at least 2 populations	80%	2998
5	9	27	1 SNP per locus, loci required to have heterozygosity <0.75 and MAF >0.05	At least 50% of individuals in at least 2 populations	80%	2996
6	9	27	1 SNP per locus	At least 85% of individuals in all 3 populations	94%	672
7	9	27	1 SNP per locus	All individuals in all 3 populations	100%	81

MAF, minor allele frequency.

Because no genome is currently available from a close relative of the Black-footed Albatross, we used BLASTN 2.2.29+ (Altschul et al. 1997) to map the consensus sequences of polymorphic loci from data set 1 to the two most complete bird genomes available online, the chicken (*Gallus gallus*) with Ensembl assembly number 4.0 (Hillier et al. 2004) and the Zebra Finch (*Taeniopygia guttata*) with Ensembl assembly number 3.2.4 (Warren et al. 2010), to understand the distribution of sequenced loci across the avian genome. Finally, we used the program KING (Manichaikul et al. 2010) to verify that we had not sampled any related individuals that could bias the analyses.

### Population differentiation

In addition to the summary statistics estimated with Stacks, we estimated additional related population genetic estimates both to confirm trends suggested by Stacks and to provide additional resolution to the demographic history. We first used STRUCTURE 2.3.4 (Pritchard et al. 2000) on data sets 1–3 to determine the number of genetic groups, or ‘clusters’ (K) that best fit the data, and assign individuals to cluster(s). STRUCTURE was run three times with each data set for *K* = 1–8 under an assumption of admixture, with 100 000 cycles of burn-in (BURNIN = 100 000) and 300 000 Markov chain Monte Carlo samples (NUMREPS = 300 000). We combined the replicate result files using CLUMPP v 1.1.2 (Jakobsson and Rosenberg 2007), implemented in STRUCTURE HARVESTER (Earl and vonHoldt 2012), and visualized the combined result file using *distruct* v. 1.1 (Rosenberg 2004). To examine population differentiation in a multivariate framework, we used R 3.0.2 to run a discriminant analysis of principal components (DAPC) on all data sets using the *adegenet* package (Jombart and Ahmed 2011). With the *hierfstat* package in R (Goudet 2005), we calculated pairwise *F*_ST_ values among the three populations for data set 1 and tested their significance by running 99 random permutations of individuals to obtain null distributions. Finally, using methods outlined in Wakeley (2008), we estimated linkage disequilibrium (*r*^2^) between sites for loci in data set 1 with more than one SNP, to determine the level of non-independence of SNPs. For these calculations, we randomly chose eight individuals from each population because an imbalanced sample size can bias the calculation of *r*^2^.

### Analysis of gene flow

For all seven data sets, we used BayeScan 2.1 (Foll and Gaggiotti 2008) to search for possible *F*_ST_ outliers among the SNPs and estimated gene flow among populations with multiple runs of the software MIGRATE 3.6.5 (Beerli and Felsenstein 2001; Beerli 2006, 2009). The MIGRATE analysis was run in Bayesian mode and assumed either nonsymmetric or symmetric migration rates between the three populations. We investigated the effect of analysing only polymorphic loci versus all loci, including invariant ones, and found little effect on the demographic estimates (Table2). To obtain MIGRATE estimates of diversity comparable to those estimated by Stacks, we assumed that each SNP was embedded in a 90-bp sequence of otherwise invariant sites. As another window into recent gene flow, we conducted population assignment tests using Genodive (Meirmans and Van Tienderen 2004), using data set 2 for this purpose (see Tables S2 and S3).

**Table 2:** RADseq run information per population (Tern, Midway, Torishima or all) for all positions (both variant and fixed), based on data set 1

Populations	All
Total number of 100-bp reads produced by the RADseq run	78 803 224
Number of loci in Stacks catalogue before filtering in *populations*	115 359
Number of loci retained in Stacks catalogue after filtering in *populations*	9760
Total number of RAD sites	879 856
Number of detected SNPs (data set completeness: 80.4%)	3455
Number of polymorphic RAD loci with ≥1 SNP	2769

Populations	Tern	Midway	Torishima
Average number of individuals per population across loci	13.9927	7.80374	13.8236
Stack depth of coverage	Mean = 180.9 SD = 150.8	Mean = 120.3 SD = 128.2	Mean = 203.2 SD = 148.3
Total number of RAD sites present in all individuals	873 713	787 400	857 790
Total number of polymorphic RAD sites present in all individuals	2647	1871	2066
Number of variant sites	3426	3154	3385
Observed heterozygosity	0.00063	0.00063	0.00059
Observed homozygosity	0.99937	0.99937	0.99941
Expected heterozygosity	0.00062	0.00060	0.00058
Expected homozygosity	0.99935	0.99936	0.99940
*π* (nucleotide diversity)	0.00065	0.00064	0.00060

SNP, single nucleotide polymorphism.

### Morphological data

We obtained morphological data from 43 museum specimens belonging to seven different museum collections (Table S4). To minimize bias due to different people performing the measurements, we took great care to describe the measuring protocol to the measurer in detail. Specimens were classified as belonging to either the Hawaiian or Japanese population, and as male or female. A specimen was assigned to a given population if it had been ringed there as a chick or caught there as an adult during the Black-footed Albatross’ breeding period (November to June). Birds caught at sea or caught at locations of interest outside of the breeding period were excluded from the analysis. The aim was to obtain measurements of wing chord, beak width, culmen length and tarsus length for all specimens, but, depending on the preparation method, certain measurements could sometimes not be taken. Sample sizes for wing chord, tarsus length, culmen length and beak width, respectively, are as follows: Japan males: 8, 9, 9, 9; Japan females: 7, 7, 8, 8; Hawaii males: 9, 8, 9, 8; and Hawaii females: 9, 10, 8, 7 (Table S4).

We used R Commander 2.0–3 to perform a factorial analysis of variance (anova) to determine whether Japanese Black-footed Albatrosses differed significantly in size from Hawaiian Black-footed Albatrosses for the different measures, controlling for sex.

## Results

### Characterization of RADseq data

We obtained a total of 78 803 224 paired-end reads, each 100 bp, across 47 individuals in the three albatross populations. The catalogue built from Stacks contained a raw total of 115 359 loci. Filtering procedures in *populations* with the criteria specific to each of the seven data sets resulted in levels of completeness ranging from 80% to 100% (Table1). After filtering in *populations* for a minimum stack depth of 30, and presence in at least 50% of the individuals of two populations, 9760 loci were retained (data set 1). From this set of loci, a total of 3455 SNPs (variable sites) were found among 2769 (28.4%) of the loci, resulting in a data matrix that was 80.4% complete across all SNPs and individuals (Figure S1). Of these 2769 loci, 2234 had only one SNP, 418 had two, 91 had three, 21 had four, and 5 had five or more. Additional information on the results based on data set 1 can be found in Table2.

**Figure 2:**
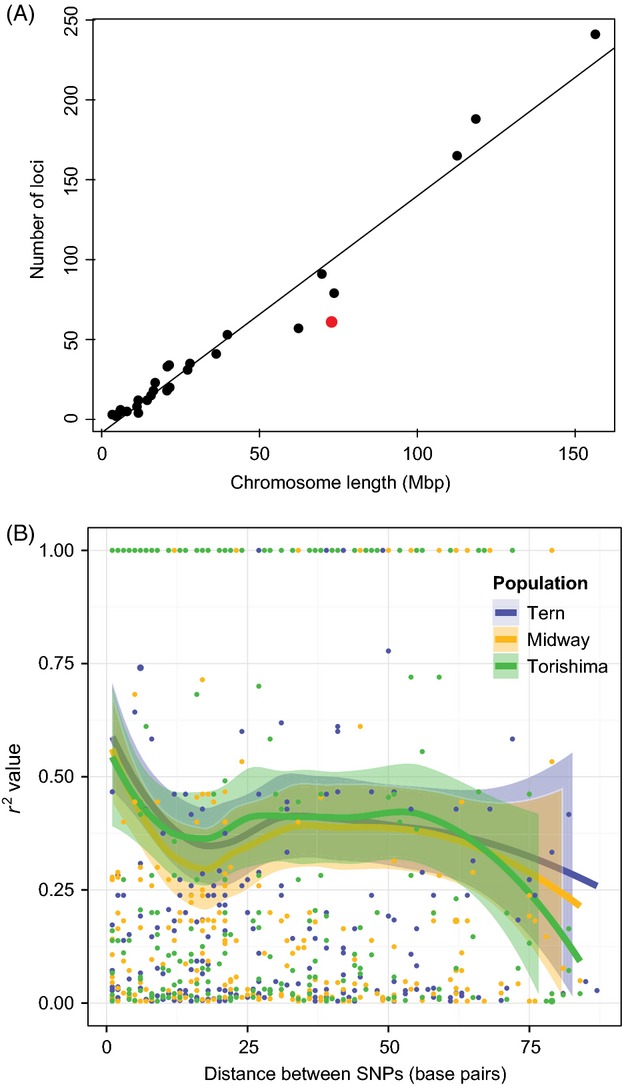
Panel A shows the chromosomal distribution of data set 1 single nucleotide polymorphisms (SNPs) as mapped to the Zebra Finch genome with an e-value <−10. Chromosome length is significantly correlated with the number of SNPs (*R*^2^ = 0.961, *P*-value <0.0001). The red dot corresponds to the *Z* chromosome loci. Panel B shows linkage disequilibrium as measured by *r*^2^ (Wakeley 2008) between pairs of SNPs found on the same RADseq locus. For eight randomly chosen individuals from each population, the LOESS smoothed *r*^2^ value (solid lines) plus 95% SE confidence intervals (Wickham 2009) are shown for each SNP distance.

When these loci were mapped to the chicken and Zebra Finch genomes, respectively, 10.8% and 16.3% mapped only once with an e-value less than −20. Of the loci, 88.8% and 80.2% did not map at all to the chicken and Zebra Finch genomes, respectively. The remaining loci (0.4% and 3.5%) mapped more than once. The higher percentage of mapped loci to the Zebra Finch genome is likely because the Black-footed Albatross and Zebra Finch share a more recent common ancestor than either does with the chicken (Hackett et al. 2008). If the e-value is relaxed to −10, 40.3% of the loci map once to the Zebra Finch genome. The number of hits per chromosome is significantly correlated with the length of the chromosome (*R*^2^ = 0.961, *P*-value <0.0001), suggesting that the SNPs are randomly distributed across the avian genome (Fig.2, panel A). There were fewer loci mapping to the Zebra Finch Z chromosome than expected given its length (Fig.2, panel A).

Linkage disequilibrium among sites within loci declined gradually with distance along the sequence for all three populations, with sites close together (<10 bp) exhibiting *r*^2^ values averaging 0.4–0.5 and sites >75 bp away exhibiting values closer to 0.2–0.3 (Fig.2, panel B). The program KING (Manichaikul et al. 2010) confirmed that we had not sampled any close relatives (results not shown). The kinship values ranged from −0.2807 to 0.0831, with 822 individual comparisons ≤0 and 80 positive comparisons. All values were below the 2nd-degree relationship cut-off value of 0.0884.

### Genetic diversity

**Figure 3:**
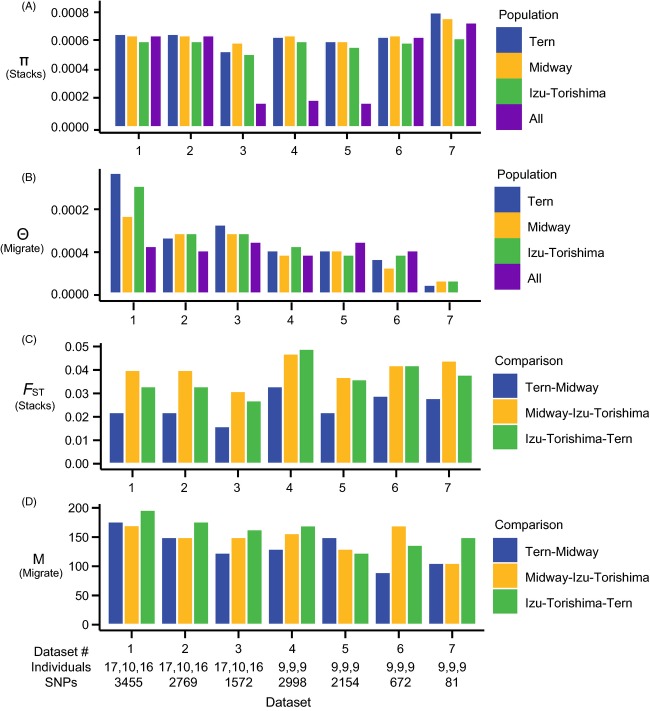
Estimates of genetic differentiation and gene flow estimated from the seven data sets listed in Tables S2 and S3. π (panel A) and θ (panel B) are reported per base pair. *F*_ST_ (panel C) was calculated in Stacks as described. M (panel D) is the estimate of m/μ in MIGRATE. m/μ is interpreted as the relative number of migration events per mutation across a given data set. When multiplied by the appropriate θ, it can be interpreted as the traditional estimate of gene flow, Nm.

**Figure 4:**
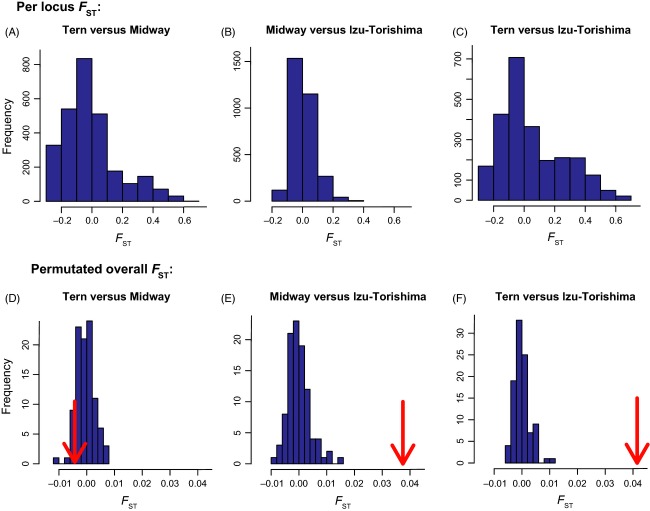
Panels A, B and C show distributions of observed per locus pairwise *F*_ST_ values calculated with data set 1 between Tern and Midway (A), Izu-Torishima and Midway (B) and Izu-Torishima and Tern (C). Panels D, E and F show pairwise *F*_ST_ values generated by 99 random permutations of all individuals compared to the pairwise population *F*_ST_ values estimated with our genetic data, given by the red arrow. Comparisons between the two Hawaiian islands, Tern and Midway (D), is not significant because it falls within the null distribution of *F*_ST_ values. *F*_ST_ values between Izu-Torishima and Midway (E) and Izu-Torishima and Tern (F) are significantly higher than expected at random.

Estimates for genetic diversity as calculated by Stacks in each population for data set 1, examining polymorphic loci only, were as follows: *π*_Tern_ = 0.165, *π*_Midway_ = 0.160 and *π*_Torishima_ = 0.152. Examining both variant and invariant loci, values varied from *π*_Torishima_ = 0.00060 to *π*_Tern_ = 0.00065 (Table S2). All other data sets also had *π* values of 0.0006, except data set 7, for which *π *= 0.0008 (Fig.3, Table S2). Pairwise *F*_ST_ values calculated for data set 1 varied between 0.022 (*F*_ST-Midway-Tern_) and 0.040 (*F*_ST-Midway-Torishima_; Table S3). Pairwise *F*_ST_ values were consistent among data sets, with *F*_ST-Midway-Tern_ always being lower than the two other *F*_ST_ values (Fig.3, Table S3). The among-locus distributions of observed pairwise *F*_ST_ values between islands in data set 1 are shown in Fig.4(panels A–C). The null distribution of *F*_ST_ values produced from permutation tests indicate that *F*_ST_ values between Torishima and Midway or Tern are significantly higher than would be expected at random, but not between Midway and Tern (Fig.4, panels D–F). BayeScan did not detect any *F*_ST_ outliers in any of the data sets, suggesting that the loci assayed in this study are likely to be evolving neutrally and that demographic processes predominantly influence the distribution of RADseq variation in this species. All in all, the different filtering procedures had very little impact on the population genetics summary statistics (Tables S2 and S3).

### Population differentiation

**Figure 5:**
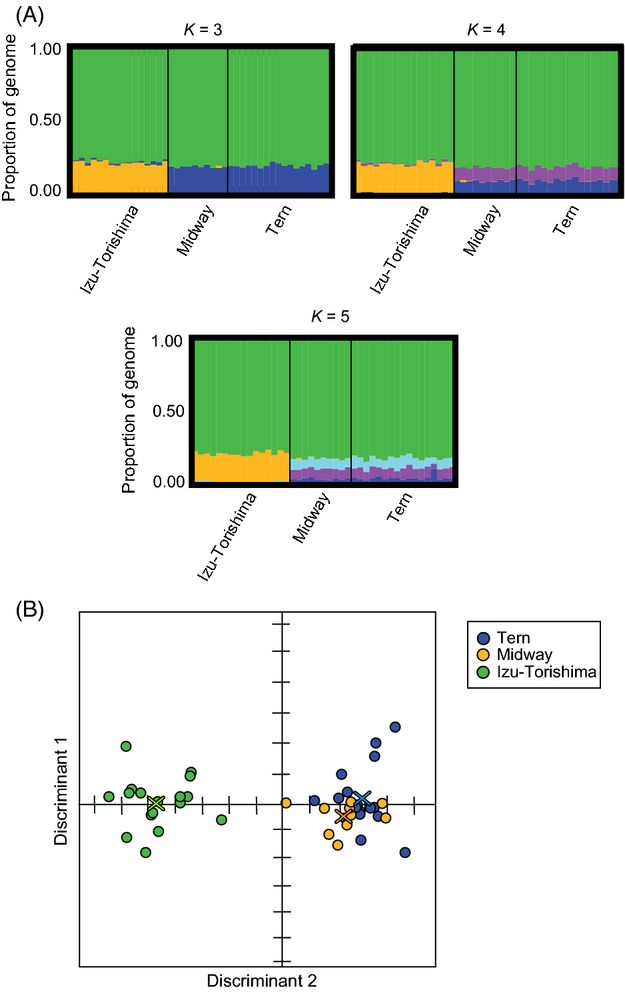
(A) STRUCTURE plots for Black-footed Albatross using RADseq data set 2 (see Table1). Models with *K* = 3–5 were the most highly supported and consistently suggest two clusters corresponding to Hawaii and Japan, albeit differentiated in only a small portion of the genome. (B) Discriminant analysis of principal component (DAPC) plot based on data set 1 indicating that Hawaiian individuals from Tern and from Midway group together, but not with individuals from Izu-Torishima in Japan.

Here, we report the STRUCTURE results for data set 2 (1 SNP per locus), because STRUCTURE can be sensitive to linked loci. The results of the other six data sets were qualitatively similar. The log-likelihood of the model was very similar across *K* = 1–5 (not shown), but maximized at *K* = 3 clusters, whereas *K* = 5 was favoured by the Delta K test (Earl and vonHoldt 2012; Fig.5A). Models with *K* = 3, 4 or 5 showed evidence of two major clusters dividing Japan and Hawaii, albeit for a small portion of the genome (Fig.5A). Despite the high amount of shared SNP variation genome-wide between Izu-Torishima and the Hawaiian islands, STRUCTURE detected more differentiation between Japan and Hawaii than between the two Hawaiian islands (Fig.5A).

For data sets 1–6, using the first three PC axes and two discriminant axes, the DAPC plot shows that individuals from Hawaii cluster together as one population, while the Japanese individuals cluster as a distinct population (Fig.5B). Eigenvalues for data set 1, which were similar across all data sets, were 321.34 for the first discriminant and 3.13 for the second, suggesting that most of the variation exists between the Japan and Hawaii populations. Because DAPC is based on a dimension reduction of the data using principal component analysis (PCA) followed by a linear discriminant analysis, the choice of the number of PCA axes to be retained at this stage needs to be evaluated. Here, the results were robust when considering different numbers of retained PC axes. Data set 7 was the only data set for which the three DAPC clusters were not clearly distinguishable; however, this is probably a result of the low total number of loci (Table1).

### Estimates of gene flow and effective population size

Using MIGRATE, estimates of genetic diversity (Θ = 4Nμ) varied depending on the model and on the data set used, ranging from 0.025 (0.00028 per bp; data set 1, Tern, symmetric model) to 0.0029 (0.00002 per bp; data set 7, Torishima, nonsymmetric model; Table S3). In general, in contrast to the results for π from Stacks, Θ decreased with the number of SNPs in the data set. However, results are consistent in that they all indicate similar levels of genetic diversity in all three populations. Across data sets and models, there was no population with consistently lower or higher estimates of Θ. Estimates of Θ for the entire species were sometimes higher but sometimes lower than estimates for individual populations, and ranged from 0.0102 to 0.0067, with a mean of 0.0088 (0.0001 per bp). Even the highest estimates of Θ suggest a low level of genetic diversity in the species as a whole.

Using standard population genetic theory for a single population at equilibrium, we estimated the effective population size of the species from our estimates of *π* and Θ, because under neutrality, both of these parameters estimate 4Nμ. Assuming a mutation rate for noncoding DNA in birds of 1.5 × 10^−9^ per site per year (Ellegren 2007) and a value of ∼18 years per generation (Niel and Lebreton 2005) yields a mutation rate of 2.7 × 10^−8^ per generation. From this value, we calculate estimates of *N* ranging from 500 to 15 881, depending on our assumptions about generation time (Table S5).

Maximum-likelihood estimates of gene flow measured on the scale of mutations (m/μ) for all data sets varied from 10.0 (data set 7; Midway-Torishima) to 210.0 (data set 7; Midway -> Torishima) (Fig.3; Table S3). Multiplying estimates of the gene flow parameter by Θ of the receiving population (or by the average of Θ in the case of symmetric migration) yielded estimates of gene flow (Nm) of 0.02–4.5 birds per generation across all pairs of islands and models assumed (Table S3). Estimates of gene flow were consistently lower for data set 7, likely a result of its small size (Table S3), and higher for data set 1. For all other data sets, all Nm values were consistently close to or >1 bird per generation. Averaging Nm for each pair of populations across all data sets and nonsymmetric models, we obtained an estimate of 1.59 birds per generation moving from Hawaii to Japan and 1.34 birds per generation moving from Japan to Hawaii, indicating highly symmetric gene flow patterns between the two colonies.

#### Population assignment tests

We performed a population assignment analysis on data set 2 using the program Genodive with 100 replicated data sets with a total of 4274 resampled individuals. The inferred population membership for each individual, inferred by calculating likelihoods coming from each population, showed strong evidence for two populations as in other analyses (not shown). All Izu-Torishima individuals were assigned to a single population, and almost all individuals from both Tern and Midway were assigned to the Midway population, with two of the Midway individuals assigned to Tern. Overall, these results corroborate the pattern of high gene flow between Japanese and Hawaiian colonies, with little differentiation within Hawaii.

### Morphological measurements

Our morphological data satisfied the necessary requirements for a factorial anova (normality of residuals, equality of population variances, independence of residuals). No statistically significant differences in size between males and females were found, except for slight differences in beak length and width: on average, males’ beaks were 1.3 mm broader (*F*_1,28_ = 8.201, *P* = 0.013) and 3.2 mm longer (*F*_1, 30_ = 9.418, *P* = 0.019) than females’. This represents a difference of 4.5% and 3.1% in width and length, respectively. Between Japan and Hawaii, the tarsus of Japanese individuals was, on average, 11.9 mm longer than that of Hawaiian individuals (*F*_1,30_ = 16.809, *P* < 0.001), representing a 12% difference in length. The wing chord of the Japanese individuals was on average 9.1 mm shorter, a 1.8% difference, although this result was not statistically significant at the 0.05 level (*F*_1,29_ = 1.804). Japanese albatrosses also had shorter beaks, by 3.2 mm on average (*F*_1,30_ = 9.444, *P* = 0.004), representing a 3.1% difference in length (Table3).

**Table 3:** Results of a factorial anova for four measurements of Black-footed Albatrosses. Effects significant at the 0.01 level are in bold

Measurement	Total sample size	Mean for all birds (mm)	Mean_Hawaii_ – Mean_Japan_ (mm)	Absolute difference of location effect (%)	Effect of location (*P*-value)	Effect of sex (*P*-value)	Interaction between sex and location
Bill width	32	30.2	−0.8	2.6	0.1151	**0.0078**	0.3470
Culmen	34	103.3	3.2	3.1	**0.0044**	**0.0045**	0.7994
Tarsus	34	98.5	−11.9	12	**0.0003**	0.2021	0.8391
Wing chord	33	494.2	9.1	1.8	0.1896	0.9207	0.4507

## Discussion

We conducted a genome-wide survey of genetic variation in Black-footed Albatrosses using RADseq, in an effort to assess the level of connectivity of colonies in Hawaii and Japan and to evaluate the implications for conservation. To study the effect on parameter estimates of different SNP filtering and individual sampling protocols, we generated seven data sets containing between ∼3500 and 81 genomewide SNPs. Our results provide further resolution and address some of the contradictions among previous studies based on mitochondrial DNA and microsatellites in this species. Overall, our results suggest high levels of gene flow among colonies within Hawaii and between Hawaiian and Japanese colonies. Although genetic differentiation between Hawaiian and Japanese colonies was detectable – the *F*_ST_ values, STRUCTURE analysis and DAPC all indicate that the Japanese and Hawaiian populations of the Black-footed Albatross are genetically differentiated, if only slightly – the overall level of differentiation was minimal. With the exception of data set 7, which, although 100% complete, had only 81 SNPs, Nm was almost always >1 between all pairs of populations, a level above which there is predicted to be no significant genetic divergence of allele frequencies in the long term (Wright 1931). On the one hand, our results confirm the suspicion (Walsh and Edwards 2005) that nuclear data might show more evidence of gene flow among colonies than mtDNA, but they also suggest that nuclear gene flow between Japanese and Hawaiian colonies is much higher than the mitochondrial gene flow measured in previous studies (Walsh and Edwards 2005; Eda et al. 2008). Our results are consistent with the recent study by Ando et al. (2014), based on 10 microsatellite markers, which found that Hawaii and Japan are detectably genetically differentiated, albeit with high levels of gene flow between Japan and Hawaii. However, whereas Ando et al. (2014) found highly asymmetrical gene flow, with most migrants apparently moving from Japan to Hawaii, our study revealed highly symmetrical levels of gene flow between the two archipelagos. Overall, the emerging picture of significant gene flow among Black-footed Albatross colonies is what one would expect for a highly vagile pelagic seabird, despite the suggestion that the species shows high philopatry (see below). However, it is well known that banding studies in birds are limited by a finite search space and challenges of resighting individuals and that apparent site fidelity is not incompatible with significant gene flow as evidenced by genetic markers (Barrowclough 1978; Koenig et al. 1996). In the case of the Black-footed Albatross, even though the species only breeds on just a few islands, it would still be difficult to detect the rare migration events, which are still significant in terms of gene flow, given the long generation time of the species (see below). Very complete data spanning many decades would be needed to measure dispersal rates accurately.

The microsatellite data from Ando et al. (2014) also suggest similar levels of genetic diversity between Japan and Hawaii, in agreement with the cytochrome *b* data from Eda et al. (2008), but differing from the mitochondrial DNA results of Walsh and Edwards (2005). Our study finds trends more similar to Eda et al. (2008): both our *π* and Θ values, calculated with Stacks and MIGRATE, respectively, suggest similar levels of diversity in all colonies. The RADseq results are indeed consistent with the suggestion that the Japanese colony, despite harbouring vastly fewer birds than those in Hawaii, possesses as much SNP variability across the genome. Indeed, the similar levels of genetic diversity when considering all individuals in a single population, as well as the uniformly low *F*_ST_ values, suggest that most of the genetic variation in the entire species can be found within any one population that we sampled. The amount of genetic variation we found was low: although few studies have been conducted with the RADseq method, we found an amount of genetic variation that is about 10 times lower than that in the human species, which is notorious for being depauperate in genetic variation (Rosenberg et al. 2002).

For all populations, our estimates of genetic diversity are likely biased downward because the RADseq approach cannot score genotypes where one chromosome is missing the relevant restriction site (Arnold et al. 2013). However, we can still compare our estimates of diversity to those of other studies using similar methodologies. Published estimates of genetic diversity in birds using the RADseq approach are rare (Harvey and Brumfield 2015), although several recent bird studies have used RADseq to study hybridization (Rheindt et al. 2013; Taylor et al. 2014a,b), and, using simulations, Harvey et al. (2015) suggested that 5000 SNPs should be adequate for estimating demographic parameters. Despite recently colonizing previously glaciated areas in North America, genetic diversity of threespine sticklebacks (Teleosti *Gasterosteus aculeatus*) was ten times that in our study (∼0.001–0.004, including all sites; Catchen et al. 2013a). Our estimates of *N*_e_, ranging 500–15881, are 4–100 times lower than the census size for this species. The estimate of generation time made by Niel and Lebreton (2005) came from an average age of first breeding of 8.6 years (Cousins and Cooper 2000) and estimates of adult survivorship and the population growth rate. The value for the mutation rate we used would have to be many times higher to account for the discrepancy between effective and census size. Although our estimates of *N*_e_ are imprecise, they reflect the low level of RADseq diversity found in this species. The downward bias in diversity incurred by the RADseq method (Arnold et al. 2013) means that our estimate of *N*_e_ is also likely downwardly biased from the true value. Still, the magnitude of the bias is likely not sufficient to reconcile the effective and census sizes in this species.

The estimates of *F*_ST_ from our study are significantly lower than that found between sister species of other birds measured with RADseq, such as closely related flycatcher and chickadee species (Rheindt et al. 2013; Taylor et al. 2014b). The BayeScan analysis did not reveal any *F*_ST_ outliers, suggesting that the loci in this study are likely to be evolving neutrally. However, despite the large number of SNPs detected in our study, the density of SNP detection by RADseq, even for a small avian genome, may not be high enough to be influenced by genomic sites under selection. Frequently, SNPs showing unusual levels of differentiation are located near genes influenced by natural selection and may be of particular use in the functional understanding of genetic variation in threatened species (Storz 2005). Seabirds are an evolutionarily old lineage and may show less evidence of ongoing natural selection than other birds. Indeed, marine vertebrates in general have been suggested to evolve slowly at genes experiencing natural selection in other groups (Slade 1992). Although our RADseq data are not fine enough to detect any signals of selection in albatross genomes, our data might hint at the possibility that the evolutionary potential, or the ability of albatrosses to evolve in response to changing environmental pressures, is relatively low, given the apparent lack of loci under natural selection as compared to other RADseq studies where outliers are routine (Catchen et al. 2013a; Moore et al. 2014; Taylor et al. 2014a). Transcriptome studies, which focus on variation in coding regions, might produce more evidence of loci under natural selection. Although our RADseq loci often mapped reliably to individual sites in the Zebra Finch genome, nearly all of these sites were in noncoding regions, which may be less subject to natural selection.

Our results are applicable to other albatross species, due to distinctive shared life-history traits that can influence evolutionary rates. For example, albatrosses have an exceptionally long life expectancy that is 20–40 times higher than that of a typical songbird. Among albatross species other than *Phoebastria nigripes*, some, such as the Shy Albatross (*Thalassarche cauta*), exhibit genetically very distinct breeding populations with little dispersal between sites (Abbott and Double 2003a,b), while others, such as the White-capped Albatross (*Thalassarche steadi;* Abbott and Double 2003a,b) and the Wandering Albatross (*Diomedea exulans*; Milot et al. 2008; but see Burg and Croxall 2004) show very little genetic structure and significant migration between populations. The Black-footed Albatross’ closest relative, the Laysan albatross (*Phoebastria immutabilis*), falls in this second category, according to a study by Young based on mitochondrial DNA (2010). Nunn and Stanley (2000) found that rates of cytochrome *b* evolution were lower in large bodied Procellariformes such as albatrosses, suggesting that slow rates of molecular evolution might be part of a general syndrome of this clade.

After evaluating the genetic differentiation between the Japanese and Hawaiian Black-footed Albatrosses, we compared these two populations morphologically. In particular, we tested the claim that Japanese individuals are smaller than their Hawaiian counterparts. Contrary to what is commonly found in the literature, we did not find Japanese Black-footed Albatrosses to be uniformly smaller than their Hawaiian counterparts. We did find some statistically significant morphological differences among geographical categories of birds (longer tarsus in Japan, longer culmen in Hawaii; Table3), but they were generally small and were not consistent in direction between Japanese and Hawaiian birds. Some of the morphological differences we found were within the range of differences that might accumulate in museum specimens over time, especially if curated under different conditions in different museums (Bjordal 1983; Kuczyński et al. 2002). Although some of the differences we found were statistically significant, the biological significance of these differences is questionable until further studies, ideally involving live birds of known age, are conducted.

In summary, our results indicate that the absolute level of genetic differentiation between the Hawaiian and Japanese Black-footed Albatross population is small, that estimated levels of gene flow are high and that individuals from the two populations do not differ markedly in size. Despite this, our study does show that the Japanese and Hawaiian populations are detectably genetically differentiated. These results illustrate the challenges faced when interpreting population dynamics estimates based on genetic data for the purpose of species management. From an evolutionary perspective, which focuses on long timescales, the two populations are unlikely to constitute separate ESUs, especially considering our estimate of gene flow, which is high enough to prevent genetic drift and population divergence (Wright 1931). This conclusion remains true despite the seeming small number of migrants per generation: >1 migrant per 18 years does not seem like high rates of exchange, yet, as a general rule, such a level would impede strong divergence in the long term. However, from a conservationist’s perspective, which focuses on shorter timescales, even a small amount of genetic differentiation could prove to be important for population management purposes, especially if two populations are demographically independent, showing, for example, different life histories, breeding schedules or population growth rates (Oostermeijer et al. 2003; Ohara et al. 2006; Angeloni et al. 2014; Palkovacs et al. 2014). Because of this possibility, and because the Hawaiian and Japanese breeding grounds are likely to be differentially affected by climate change for geographical reasons (Storlazzi et al. 2013), we suggest that the Japanese and Hawaiian populations of the Black-footed Albatross be considered separate biological MUs. A more widespread species is inherently more resilient to extinction; hence, it is worthwhile to preserve as much of a species’ range as possible, given predicted threats such as climate change and rising sea levels.

Nevertheless, because of low levels of genetic and morphological differentiation, we reject our initial hypothesis that the Japanese population is sufficiently differentiated to deserve subspecific status, and support the ACAP’s decision not to consider the Japanese population a separate subspecies of the Black-footed Albatross. Radio telemetry or banding data on individual birds’ movements between the different Black-footed Albatross colonies would help refine our understanding of gene flow in this species and patterns of philopatry. Between 1980 and 2007, 13 854 chicks were banded on Tern Island, and recaptures of breeding birds in Hawaii started in 1992 (Véran et al. 2007). Remarkably, however, only two birds known to have been banded as chicks in Japan have been observed to be breeding in Midway, Hawaii, in 2014 and 2015 (K. Yoshiyashu and S. Doell, pers. comm.). Apparently, these two migratory events are the only ones recorded thus far (B. Flint, pers. comm.). Over time, exchanges of banding and recapture data between Hawaiian and Japanese researchers could allow for detection of these rare migration events to estimate direct migration rates between the two populations, but this would require a highly concerted and large-scale effort. These data, in concert with genetic analysis of adaptively evolving loci, would provide insight into the mechanisms through which the subtle genetic and morphological differences within the Black-footed Albatross arise.

## Data archiving statement

Data available from the Dryad Digital Repository: http://dx.doi.org/10.5061/dryad.71gt8.

## Supporting Information

**Figure S1.** RADseq data set completeness plot for data set 1.

**Table S1.** List of specimens used in the genetic study.

**Table S2.** Estimates of genetic diversity using different data filtering criteria.

**Table S3.** Estimates of *F*_ST_ and migration rates using different data filtering criteria.

**Table S4.** List of specimens used in the morphological study.

**Table S5**. Estimates of effective population size from RADseq data using a range of estimated generation times.
