# Supplementary figures and images for: Morphological and genomic comparisons of Hawaiian and Japanese Black-footed Albatrosses (Phoebastria nigripes) using double digest RADseq: implications for conservation
Source: Evol Appl. 2015 Jun 13;8(7):662–78. doi: 10.1111/eva.12274 (PMC4516419; doi:10.1111/eva.12274)

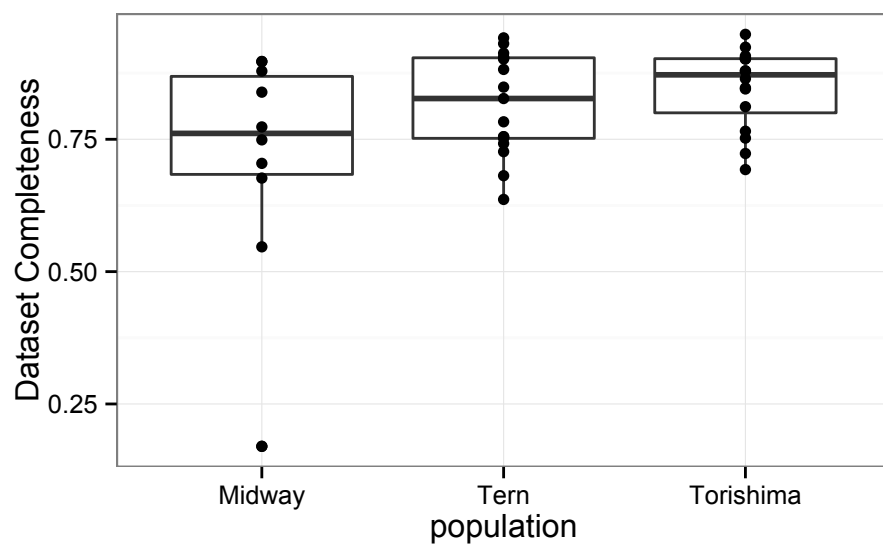

Supplementary Figure 1.

Supplement: Supplementary file 1 [file eva0008-0662-sd1.pdf]
