# Supplementary material for: Morphological and genomic comparisons of Hawaiian and Japanese Black-footed Albatrosses (Phoebastria nigripes) using double digest RADseq: implications for conservation
Source: Evol Appl. 2015 Jun 13;8(7):662–78. doi: 10.1111/eva.12274 (PMC4516419; doi:10.1111/eva.12274)
Supplement: Supplementary file 2 [file eva0008-0662-sd2.docx]

**Supplementary Table 1.** Specimens used for Rad-sequencing.

| **Lab Sample Number** | **Collecting Institution** | **Collection Tissue Number** | **Country/State** | **Island** | **Date of Sample Collection** |
| --- | --- | --- | --- | --- | --- |
| 4 | Burke | HEW 242 | Hawaii | Tern | 2000 |
| 8 | Edwards Lab (Harvard) | BFAL 676 | Hawaii | Midway | 1994 |
| 9 | Yamashina Institute for Ornithology | 3 | Japan | Toshima | 1996 |
| 41 | Edwards Lab (Harvard) | BFAL 692 | Hawaii | Midway | 1994 |
| 36 | Burke | HEW 082 | Hawaii | Tern | 2000 |
| 50 | Yamashina Institute for Ornithology | 6 | Japan | Toshima | 1996 |
| 40 | Burke | HEW 071 | Hawaii | Tern | 2000 |
| 35 | Burke | HEW 070 | Hawaii | Tern | 2000 |
| 52 | Edwards Lab (Harvard) | BFAL 675 | Hawaii | Midway | 1994 |
| 51 | Edwards Lab (Harvard) | BFAL 693 | Hawaii | Midway | 1994 |
| 38 | Edwards Lab (Harvard) | BFAL 689 | Hawaii | Midway | 1994 |
| 49 | Edwards Lab (Harvard) | BFAL 677 | Hawaii | Midway | 1994 |
| 46 | Yamashina Institute for Ornithology | 15 | Japan | Toshima | 1996 |
| 45 | Burke | HEW 255 | Hawaii | Tern | 2000 |
| 44 | Burke | HEW 256 | Hawaii | Tern | 2000 |
| 33 | Yamashina Institute for Ornithology | 17 | Japan | Toshima | 1996 |
| 20 | Yamashina Institute for Ornithology | 1 | Japan | Toshima | 1996 |
| 3 | Burke | HEW 234 | Hawaii | Tern | 2000 |
| 32 | Burke | HEW 069 | Hawaii | Tern | 2000 |
| 43 | Yamashina Institute for Ornithology | 18 | Japan | Toshima | 1996 |
| 42 | Burke | HEW 067 | Hawaii | Tern | 2000 |
| 48 | Yamashina Institute for Ornithology | 10 | Japan | Toshima | 1996 |
| 26 | Yamashina Institute for Ornithology | 13 | Japan | Toshima | 1996 |
| 30 | Yamashina Institute for Ornithology | 16 | Japan | Toshima | 1996 |
| 17 | Burke | HEW 072 | Hawaii | Tern | 2000 |
| 23 | Burke | HEW 240 | Hawaii | Tern | 2000 |
| 37 | Burke | HEW 061 | Hawaii | Tern | 2000 |
| 39 | Yamashina Institute for Ornithology | 12 | Japan | Toshima | 1996 |
| 18 | Yamashina Institute for Ornithology | 4 | Japan | Toshima | 1996 |
| 6 | Edwards Lab (Harvard) | BFAL 700 | Hawaii | Midway | 1994 |
| 24 | Burke | HEW 052 | Hawaii | Tern | 2000 |
| 29 | Yamashina Institute for Ornithology | 2 | Japan | Toshima | 1996 |
| 22 | Edwards Lab (Harvard) | BFAL 674 | Hawaii | Midway | 1994 |
| 34 | Edwards Lab (Harvard) | BFAL 680 | Hawaii | Midway | 1994 |
| 11 | Edwards Lab (Harvard) | BFAL 698 | Hawaii | Midway | 1994 |
| 27 | Yamashina Institute for Ornithology | 8 | Japan | Toshima | 1996 |
| 10 | Burke | HEW 235 | Hawaii | Tern | 2000 |
| 21 | Yamashina Institute for Ornithology | 5 | Japan | Toshima | 1996 |
| 16 | Edwards Lab (Harvard) | BFAL 691 | Hawaii | Midway | 1994 |
| 28 | Burke | HEW 112 | Hawaii | Tern | 2000 |
| 25 | Yamashina Institute for Ornithology | 19 | Japan | Toshima | 1996 |
| 31 | Yamashina Institute for Ornithology | 14 | Japan | Toshima | 1996 |
| 19 | Yamashina Institute for Ornithology | 20 | Japan | Toshima | 1996 |
| 14 | Burke | HEW 077 | Hawaii | Tern | 2000 |
| 47 | Yamashina Institute for Ornithology | 7 | Japan | Toshima | 1996 |
| 15 | Burke | HEW 182 | Hawaii | Tern | 2000 |
| 13 | Burke | HEW 236 | Hawaii | Tern | 2000 |
