# Supplementary material for: Morphological and genomic comparisons of Hawaiian and Japanese Black-footed Albatrosses (Phoebastria nigripes) using double digest RADseq: implications for conservation
Source: Evol Appl. 2015 Jun 13;8(7):662–78. doi: 10.1111/eva.12274 (PMC4516419; doi:10.1111/eva.12274)
Supplement: Supplementary file 5 [file eva0008-0662-sd5.docx]

**Supplementary Table 4.** Specimens used in the morphological analyses.

| **Individual Number** | **Museum Collection** | **Collection ID** | **Location** | **Sex** | **Date Collected in the Field** |
| --- | --- | --- | --- | --- | --- |
| 1 | Harvard MCZ | ornithology300355 | Hawaii | male | 18/03/1905 |
| 2 | Harvard MCZ | ornithology300356 | Hawaii | male | 21/03/1905 |
| 3 | Harvard MCZ | ornithology251010 | Japan | female | 20/01/1911 |
| 4 | Harvard MCZ | ornithology251011 | Japan | male | 29/10/1914 |
| 5 | Harvard MCZ | ornithology276781 | Japan | female | 05/04/1949 |
| 6 | Harvard MCZ | ornithology276780 | Japan | male | 05/04/1949 |
| 7 | Harvard MCZ | ornithology334795 | Hawaii | female | 18/03/1905 |
| 8 | Bishop Museum | BBM1005 | Hawaii | male | 11/04/1946 |
| 9 | Bishop Museum | BPBM178495 | Hawaii | female | 18/12/1991 |
| 10 | Bishop Museum | BPBM178580 | Hawaii | female | 27/03/1992 |
| 11 | Bishop Museum | BPBM179943 | Hawaii | male | 14/01/1994 |
| 12 | American Museum of Natural History | 193255 | Hawaii | NA | 01/12/1936 |
| 13 | American Museum of Natural History | 193256 | Hawaii | male | 01/12/1936 |
| 14 | American Museum of Natural History | 839627 | Hawaii | male | 01/02/2013 |
| 15 | Los Angeles County Museum | 115139 | Hawaii | male | NA |
| 16 | Los Angeles County Museum | 115140 | Hawaii | female | NA |
| 17 | Los Angeles County Museum | 115141 | Hawaii | female | NA |
| 18 | Los Angeles County Museum | 115142 | Hawaii | female | NA |
| 19 | Los Angeles County Museum | JMD169 | Hawaii | female | 01/04/2008 |
| 20 | Los Angeles County Museum | SAK139 | Hawaii | male | 01/04/2008 |
| 21 | Yamashina Institute for Ornithology | 00365 | Japan | female | 02/1930 |
| 22 | Yamashina Institute for Ornithology | 00370 | Japan | female | 01/1930 |
| 23 | Yamashina Institute for Ornithology | 00371 | Japan | female | 06/1926 |
| 24 | Yamashina Institute for Ornithology | 00374 | Japan | female | 04/1932 |
| 25 | Yamashina Institute for Ornithology | 00378 | Japan | female | 02/1930 |
| 26 | Yamashina Institute for Ornithology | 00394 | Japan | female | 02/1886 |
| 27 | Yamashina Institute for Ornithology | 00364 | Japan | male | 01/1932 |
| 28 | Yamashina Institute for Ornithology | 00373 | Japan | male | NA |
| 29 | Yamashina Institute for Ornithology | 00375 | Japan | male | 04/1932 |
| 30 | Yamashina Institute for Ornithology | 00377 | Japan | male | 02/1884 |
| 31 | Yamashina Institute for Ornithology | 00379 | Japan | male | 05/1931 |
| 32 | Yamashina Institute for Ornithology | 00393 | Japan | male | 1993 |
| 33 | Yamashina Institute for Ornithology | 63917 | Japan | male | 02/2006 |
| 34 | Yamashina Institute for Ornithology | 00363 | Japan | NA | 01/1931 |
| 35 | Yamashina Institute for Ornithology | 00367 | Japan | NA | NA |
| 36 | Yamashina Institute for Ornithology | 00368 | Japan | NA | 05/1900 |
| 37 | Yamashina Institute for Ornithology | 00369 | Japan | NA | NA |
| 38 | Yamashina Institute for Ornithology | 00372 | Japan | NA | 02/1936 |
| 39 | Santa Barbara Museum of Natural History | 9332 | Hawaii | male | 02/1991 |
| 40 | Field Museum | 156076 | Hawaii | male | 02/1913 |
| 41 | Field Museum | 188889 | Hawaii | female | 02/1013 |
| 42 | Field Museum | 354985 | Hawaii | female | 01/1964 |
| 43 | Field Museum | 487635 | Hawaii | female | 02/2007 |
