# Supplementary material for: Morphological and genomic comparisons of Hawaiian and Japanese Black-footed Albatrosses (Phoebastria nigripes) using double digest RADseq: implications for conservation
Source: Evol Appl. 2015 Jun 13;8(7):662–78. doi: 10.1111/eva.12274 (PMC4516419; doi:10.1111/eva.12274)
Supplement: Supplementary file 6 [file eva0008-0662-sd6.docx]

**Supplementary Table 5.** Estimates of effective population size from RADseq data using a range of estimated generation times.

|  |  | **Estimates of effective population size (N_e_), using various generation times*** | | | |
| --- | --- | --- | --- | --- | --- |
| **Colony** | π (Stacks)^#^ in red; θ per bp (Migrate)^+^: lowest estimate in blue, highest estimate in black | Minimum age at first breeding: 7 years | 17.09 years | 18 years | 20 years |
| **Tern** | 0.000642  0.00007  0.00028 | 15286  1667  6667 | 6261  683  2731 | 5944  648  2593 | 5350  583  2333 |
| **Midway** | 0.000646  0.00006  0.00018 | 15381  1429  4286 | 6300  585  1755 | 5981  556  1667 | 5383  500  1500 |
| **Torishima** | 0.000582  0.00007  0.00025 | 13857  1667  5952 | 5676  683  2438 | 5389  648  2315 | 4850  583  2083 |
| **All colonies** | 0.000667  0.00007  0.00011 | 15881  1667  2619 | 6505  683  1073 | 6176  648  1019 | 5558  583  917 |

# Values of π are the mean values for all datasets from Stacks (see Supplementary Table 2).

+Values of θ taken from the estimates from Migrate (see Supplementary Table 2, except dataset 7).
*All estimates assume a neutral mutation rate of 1.5 X 10-9 per site per year. See main text for further details. The range of generation times is taken from values published in Cooper and Cousins (2000) and Niel and Lebreton (2005).
